# Use and Conservation of Medicinal Plants by Indigenous People of Gozamin Wereda, East Gojjam Zone of Amhara Region, Ethiopia: An Ethnobotanical Approach

**DOI:** 10.1155/2018/2973513

**Published:** 2018-03-19

**Authors:** Nigussie Amsalu, Yilkal Bezie, Mulugeta Fentahun, Addisu Alemayehu, Gashaw Amsalu

**Affiliations:** ^1^Department of Biology, Debre Markos University, Debre Markos, Ethiopia; ^2^Department of Biotechnology, Debre Markos University, Debre Markos, Ethiopia; ^3^Department of Chemistry, Debre Markos University, Debre Markos, Ethiopia

## Abstract

An ethnobotanical study of medicinal plants (MPs) used by the local community has been carried out from January 5, 2014, to February 15, 2015, in Gozamin Wereda of East Gojjam Zone, Ethiopia. The purpose of this study was to identify and document the use and conservation of MPs, along with indigenous knowledge of Gozamin community. Data were collected using semistructured interview, field observation, and focus group discussions. The collected data were assessed quantitatively using fidelity level, Jaccard's Coefficient of Similarity, paired comparisons, direct matrix, and preference rankings. In total 93 MPs distributed under 51 families and 87 genera were identified and for each taxon a local name (Amharic) was documented. Asteraceae with 9 (9.68%) species and Solanaceae with 7 (7.53%) species were families represented by more species in the study area. Out of these MPs collected, 80 plant species were used for the treatment of human ailments, 24 species were used against livestock diseases, and 11 common plant species were listed in both cases. The most frequently used plant parts were the leaves followed by the roots. The major threats to MPs in the study area were agricultural expansion, overgrazing, fire wood collection, mining, and cutting down trees for construction and furniture. Therefore, there is a need for appropriate in situ and ex situ conservation measures.

## 1. Introduction

Findings indicated that Ethiopia is one of the top 25 biodiversity-rich countries in the world and hosts two of the world's 34 biodiversity hotspots, namely, the Eastern Afromontane and the Horn of Africa hotspots [1]. It is also among the countries in the Horn of Africa regarded as major center of diversity and endemism for several plant species [2]. The diverse topography gave rise to a wide range of altitude and other environmental factors that resulted in diversity of life [2–6]. Thus, the country is a land not only of highly varied landscapes and hence flora and fauna, but also of multiplicity of ethnic groups with complex cultural diversity [4].

Ethiopia has diverse medicinal flora distributed in different vegetation types. About 1,000 medicinal plants (MPs) have been identified and documented [7]. The country is also ethnobotanically rich as there are diverse ethnic groups with diverse cultures. The knowledge and skills used in medication have been held by certain families and have passed down orally from parent to children, often a much loved one [4]. For most parts of the centuries, medicinal use knowledge and practices remained oral without documentation. Documentation of those plants used as traditional medicines is needed so that the knowledge can be preserved and utilized sustainably.

The use of traditional medicine is still widespread in Ethiopia, as well as its acceptability, availability, and popularity no doubt since about 90% of the populations use it for healthcare needs [8]. However, according to Giday and Ameni [9], loss of knowledge has been aggravated by the expansion of modern education, which has made the younger generation underestimates its traditional value. Similar to elsewhere in the country, people living in Gozamin Wereda (district) have traditional practices which have passed from generation to generation in order to treat both humans and livestock ailments. The human tendency to categorize and organize knowledge and experience is universal and that emic classification system throughout the world shows certain structural similarities [10].

In spite of the vast role of ethnobotanical contributions of MPs in the primary healthcare, limited works have so far been done in the country so as to document and enhance associated indigenous knowledge (IK) [4, 11–16]. This study has therefore been initiated to study and document plants used in the traditional medical practices of the people of Gozamin Wereda together with ethnobotanical and ethnomedicinal knowledge and practices.

Like all other parts of the country majority of the people of the study Wereda used herbal medicines for a long time to treat human and livestock ailments. Still now the dependence on this medicine is continuing because of its acceptability, accessibility, and affordability. The present study aimed at identification and documentation of MPs and associated knowledge of using, managing, and conserving MPs by the community of Gozamin Wereda which also becomes useful in the introduction of alternative resource management like in situ conservation systems that involve local people which is an urgent task for the study area where its natural vegetation is lost rapidly.

The study also aspires to identify and record the use of medicinal plant species for purposes other than their medicine. Besides, scanty attempts have been made to identify factors associated with their collection, threats, utilization, and use of the plants for the communities. Thus, the research fills this gap by documenting the wealth of indigenous knowledge and understanding the corresponding drivers of this knowledge related to management and conservation of medicinal plants used to treat human and livestock ailments in study area.

## 2. Materials and Methods

### 2.1. Description of the Study Area

#### 2.1.1. Location, Demography, and Boundary

Gozamin Wereda is one of the 18 Weredas in East Gojjam Zone and 151 Weredas in Amhara National Regional State [17]. The relative location of the Wereda is 300 km away from the capital city of the country, Addis Ababa, and 260 km from Bahir Dar, the regional capital city. This Wereda is found almost mid-way from Addis Ababa to Bahir Dar. The Wereda is bounded by Senan Wereda in the North, Baso Liben Wereda and Oromia National Regional State in the South, Aneded and Debay Tilatgen Wereda to the East, and Machakle and Debre Elias Wereda in the West [17].

The total population of the Wereda has an estimated size of 173,300 of which 87,178 are males and 86,122 are females: the highest population (80,294) is in the age range of 0–14 whereas the lowest one is above the age of 60 which accounted for 7,837 in number [17]. 2094 (1.34%) of the population are urban dwellers which is less than the zonal average of 11.2%. Moreover with an estimated area of 1,218.07 square kilometers, it has an estimated population density of 128.3 people per square kilometer which is also less than the zonal average of 179.96, East Gojjam Zone Office of Finance and Economic Development [17]. The ethnicity of the Wereda population is Amhara and Amharic is every body's language.

### 2.2. Geomorphology and Vegetation Type

The topography of the Wereda is characterized by the three major agroclimatic zones within altitude range of 500–3500 meters above sea level: temperate (Dega) (35.55%), subtropical (Woina Dega) (41.41%), and tropical (Kolla) (15.72%). The Wereda relief in percentage is given as plateau 45%, mountain 48%, and valley 7%. With respect to soil type, the majority of the Wereda soil belongs to vertisol and lithosol [17].

The vegetation type of Gozamin could be Dry Evergreen Afromontane Forest. According to Friis et al. [18], Dry Evergreen Afromontane Forest (DAF) and grassland complex occurs in areas between 1800 and 3000 meters of altitude with rainfall < 1700 mm. Different authors also indicated the subtypes of DAF, namely, undifferentiated Afromontane Forest; dry single-dominant Afromontane Forest of the Ethiopian highlands; Afromontane woodland, wood grassland and grassland, and transition between Afromontane vegetation and* Acacia-Commiphora *Bushland on the Eastern Escarpments. The natural vegetation of the study area is dominated by remnant trees like* Juniperus procera, Hagenia abyssinica, Podocarpus falcatus, Acacia abyssinica, Cordia africana, Ficus sycomorus, Erythrina brucei, Eucalyptus camaldulensis, Eucalyptus globulus*, and others. Plantations of these trees are observed on farm lands and home-gardens in Wereda [17].

There is also an Afroalpine belt as long as the Wereda includes a particular subdistrict (Kebele) known as Gedellamma which is in the Choke mountain system. It is clearly indicated that mountains are virtually devoid of vegetation and the only few woody plant species observed are moorland types sparsely covered with Giant Lobelia* (Lobelia rhynchopetalum)*, lady's mantle* (Alchemilla humania)*, Guassa grass* (Festuca *spp.), and other grasses. The woody plant cover includes* Erica arborea* and Giant St. John's wort* (Hypericum revolutum)* [19].

The rainfall pattern is unimodal, stretching from May to September. The highest monthly rainfalls were record in July, 284.67 mm, and August, 298.03 mm, and the lowest ones were observed in January (14.52 mm) and February (10.6 mm). The mean annual rain fall distribution is 1342 mm. The mean annual maximum and minimum temperature records of the study site were 26°C and 8.6°C, respectively. Rainy months are blackened and dry months are dotted as indicated in Figure 2.

### 2.3. Health

The most important animal diseases in Gozamin Wereda include bacterial infections (black leg, pasteurellosis, anthrax, mastitis, fowl typhoid, and avian salmonellosis), endoparasites (fascioliasis, paramphistomiasis, and strongyloidiasis), ectoparasites (ticks, mites, lice, and insect flies), viral infections (rabies, African horse sickness, and foot and mouth diseases), and protozoal infections (trypanosomiasis, babesiasis, and coccidiosis) [17]. On the other hand, it was also reported that the most common human diseases include tuberculosis, malaria, diarrhea, trachoma, helminths, skin diseases, typhoid, eye diseases, and upper respiratory tract.

### 2.4. Reconnaissance Survey

Reconnaissance survey was conducted from September 23 to 30, 2013, in the study Wereda in order to obtain an impression about medicinal plants, topography, distribution of plants, and identification of sampling sites. During the survey 12 representative Kebeles (subdistricts) were selected. Local administrators were chosen as key resource persons in providing information in their respective Kebeles. During the survey, general information about the Wereda was also obtained.

### 2.5. Site Selection

**Figure 1:**
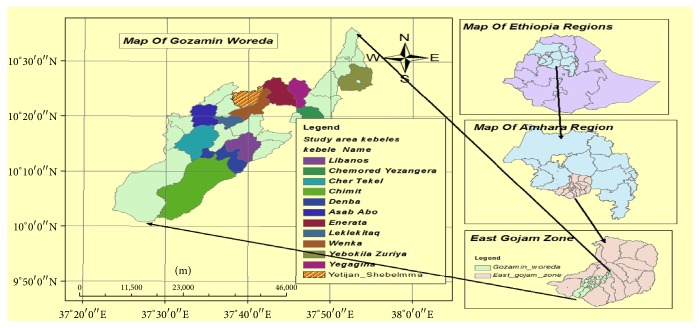
Map of Ethiopia showing location of the study area.

**Figure 2:**
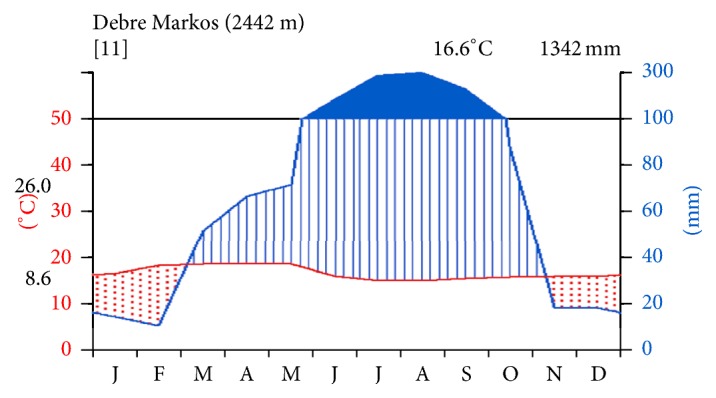
Climadiagrams of the study area from 2005 to 2014 at Debre Markos Station (data source: National Meteorological Agency).

Purposively twelve Kebeles were selected out of 26 Kebeles of the Wereda for ethnobotanical data collection based on the availability of traditional medicinal practitioners present, identified with the assistance of the Kebele administration leaders, elders, religious leaders, and others who have information about traditional healers. Those Kebeles which have almost the same agroclimatic conditions as other neighboring Kebeles were not included in the study and this is also additional factor for site selection. The study Kebeles were Libanos, Chimbord Yezangera, Cher Tekel, Denba, Asab Abo, Enerata, Lekilekita, Wenka, Chimit, Yegagina, Yebokila Zurya, and Yetijan-Shebelmma (Figure 1).

### 2.6. Selection of Informants

A total of 100 informants (88 males and 12 females) were selected from the representative Kebeles. Representative common informants and knowledgeable traditional medicine practitioners (key informants) of Gozamin Wereda were selected using random and purposive sampling approaches, respectively, following Martin [20].

Out of 100 informants, 80 general informants were sampled during random visits in the study Kebeles by asking every individual in the house or working fields or wherever they were. On the other hand, the remaining 20 key informants out of the total were selected purposively and systematically. In other words, this was based on comments and recommendations from the religious leaders, elders, Kebele administrators, students, and personal observation of the researchers from the community group. The selection of key informants was also based on the quality of explanations that particular informants gave during the interview. Local healers automatically qualified as key informants being traditional experts who are custodians of IK on the use of traditional MPs. Thus, key informants were identified, later interviewed, and followed for further details.

### 2.7. Data Sources

Ethnobotanical data were obtained from both primary and secondary sources. The primary sources were interview, focus group discussion, and observation in the field of the study area, where as the secondary data sources were various and relevant literature review related to the present study. A piece of other information was also collected from the Wereda of different offices.

### 2.8. Ethnobotanical Data Collection Techniques

The study has been carried out by interviewing the knowledgeable informants in different villages under various sites from January 5, 2014, to February 15, 2015, in Gozamin Wereda, East Gojjam Zone of Amhara Region Ethiopia. Ethnobotanical techniques were employed to collect data on knowledge and management of traditional MPs used by the local people in the study area. The methods used for ethnobotanical data collection were semistructured interviews following Martin [20] and Cotton [10], informant consensus, field observation, and group discussion as described below.

#### 2.8.1. Semistructured Interviews 

A list of questions was prepared that was covered in discussion with the informants in a particular order. The interview was guided to cover the key topics on the checklist while leaving room to pursue any relevant subject brought up by the interviewee. All of the interviews were held in Amharic, the language of the local people by the researcher. The place and the time for discussion were set based on the interest of the informants. The status of all the MPs was recorded as abundant, less abundant, rare, or very rare as per healer perception during the semistructured interviews.

#### 2.8.2. Field Observation

Field observations were carried out with local people, guidance, interviewed informants, and students. Habitat, habit, abundance, and distribution of plants were recorded in the given area.

#### 2.8.3. Focus Group Discussion

Short, brief, and precise group discussions were made with informants regarding the MPs in the study sites. Information on local names of the plants, their medicinal uses, methods of preparation, mode of administration, disease conditions, IK on MPs, and threats to plants, conservation and management of plants, and related data were recorded.

### 2.9. Voucher Specimen Collection

Plants used for herbal remedies were collected by a team comprising a botanist, biotechnologist, microbiologist, chemist, and parasitologist from Debre Markos University (DMU). The voucher specimens were collected at the spot during guided field walk, numbered, pressed, dried, and deep frozen for identification. Determination of specimens was carried out both in the field and in the herbarium. Then after that, nomenclature was determined using Flora of Ethiopia and Eritrea, other available taxonomic literatures, and visual comparison with authenticated herbarium specimens. Finally, the voucher specimens were kept at Debre Markos University Mini-Herbarium to aid confirmation of plant identity.

The information gathered included vernacular name of plant, species, ailments they cure, part used, route of administration, method of preparation, dose, and duration levels of herbal administration. Therefore, this is the starting point of documenting the flora of Gojjam in general and Gozamin Wereda of East Gojjam Zone in particular.

### 2.10. Ethnobotanical Data Analysis

#### 2.10.1. Preference Ranking

Preference ranking was included in predesigned semistructured interview items. It was conducted following Martin [20] for six MPs in treating diseases. The key informants were selected to identify the best preferred MPs for the treatment of ailments based on their personal preference or perceived degree of importance in the community. They were informed to assign the highest value (5) for the most preferred plant species and the lowest value (1) for the least preferred ones. Finally, the values were summed up and the ranks given to each plant.

#### 2.10.2. Direct Matrix Ranking

Direct matrix ranking was conducted following Cotton [10], on six multipurpose MPs. Each key informant assigned items by considering several attributes one at a time, using numerical scale in which the highest number is equal to the most preferred item whereas the lowest one is equal to the least preferred one. Then the informants rated their preferences. Direct matrix ranking can also be done as a group exercise in which participants reach consensus on the ranking of each item or vote according to their individual assessments [20].

#### 2.10.3. Paired Comparison

After having identified five most important MPs based on their medicinal values as perceived by the informants, the paired comparisons were employed following Martin [20]. Paired comparisons on the five most effective plants in treating health problems were conducted using random number table and flipping coins.

#### 2.10.4. Jaccard's Coefficient of Similarity

This can be used to determine the similarity in species composition of the study area with other study areas done by other researchers. Jaccard's Coefficient of Similarity (JCS) was used so as to assess plant species composition similarity, among seven different Weredas. It was computed between the present study area and other areas, which were studied by other authors in different parts of the country. JCS was calculated following Kent and Coker [21].

#### 2.10.5. Fidelity Level (FL)

Because many plant species may be used in the same use category, we needed to determine the most preferred species used for the treatment of a particular ailment, and we did so by calculating fidelity levels (FL) [22]: FL = (1/4)*Np*/*N*, where *Np* is the number of use-reports cited for a given species for a particular ailment, *N* is the total number of use-reports cited for any given species. High FL values (near 100%) are obtained for plants for which almost all use-reports refer to the same method of use (i.e., the plants were considered the most preferred species for a particular ailment category), whereas low FL are obtained for plants that are reused for many different purposes.

## 3. Results and Discussion

### 3.1. Summary of Informants

**Figure 3:**
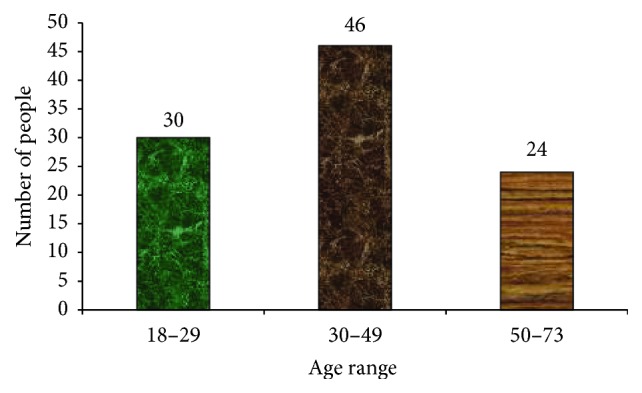
Age range of informant.

A total of 100 herbalists from the study sites were interviewed and as a result ninety-three (93) plant species belonging to 51 plant families and 87 genera were identified for the management of both human and livestock ailments (Figure 3). The present study has shown that the people at various age groups in Gozamin Wereda have a very good knowledge base on herbal remedy for primary healthcare. Traditional healers (both males and females) still rely largely on naturally growing species in their locality.

### 3.2. Age and Sex of Informants

The informants were local residents aged between 18–73 years (Figure 3). Most of traditional healers range within the age group of 30–49 years, 46 (46%). They are within this range because it is at this stage whereby they have completed necessary rites of passage, notably initiation school. These are followed by those who are around 18–29 years of age, 30 (30%), who also constitute a reasonable number; thus in most cases these are newly inaugurated traditional healers. The remaining 24 (24%) of informants were between the ages of 50 and 73 (Figure 3). Although the age groups 50–73 constitute lower percentage (24), these groups are highly trusted by the community due to experience they have acquired and the number of traditional healers they have trained. Thus, at this age their decisions are credible based on their maturity level. Medicinal plant knowledge difference among age groups was also reported in Cotton [10].

Regarding sex, 88 (88%) of the total informants were males whereas the remaining 12 (12%) were reported as females (Table 1). In the case of knowledge difference between the two sexes, females tend to mention little number of MPs as compared to males. At this point, because of cultural influence, it is understandable that females were not allowed to go out of home; they look after babies and work at home. Similar results were reported in Megersa et al. [23], Kefalew et al. [24], and Chekole et al. [25] mentioning few numbers of plants by females in our study area.

### 3.3. Educational Level and Marital Status of the Informants

The educational status of the informants showed that most of them were literate, that is, having modern education (diploma holders, students, and those able to read and write) accounting for 64 (64%) followed by the uneducated ones but traditionally knowledgeable informants accounted for 19 (19%) and the least number of respondents was reported as church education attendants, 17 (17%) (Table 1). On the other hand, the marital status of the informants indicated that most of them were married people accounting for 66 (66%) followed by the unmarried ones, 30 (30%), and others were divorced, reported as 4 (4%) (Table 1). The interviewed informants were included from married, unmarried, and divorced people and people of different educational level. This enabled the researchers to come across the ethnobotanical information of the community from different groups. Similar results were also reported by Yineger et al. [26].

### 3.4. Traditional Knowledge Transfer

The most important way of transfer of IK on types of use of MPs, mode of preparations, way of administration, traditional idea of illnesses, and methods of diagnosis and treatment among indigenous herbal practitioners of Gozamin community was by word of mouth to a family member. The finding further indicated that the present knowledge transfer system followed the inheritance based transfer system where most traditional healers pass their knowledge to the elder/eldest/son/daughter. The selection of the elect was based upon his/her good conduct and ability to keep the secret with regard to the ethnobotanical plant use knowledge. The knowledge transfer system is bounded by traditional rules and can only happen through cultural ceremony. Most of the healers confirmed that, during transfer of the knowledge, they also received obligation. Similarly it was also reported that, at family level, it is restricted to the elders (men and women), followed by elder son or daughter or their trustworthy person when the mother or the father is getting old or near to die [14, 27, 28].

### 3.5. Medicinal Plants in Gozamin Wereda

#### 3.5.1. Plants Used to Treat Human and Livestock Ailments

In the current study, ninety-three medicinal plant species distributed across 51 families and 87 genera were collected and documented from the study site (Tables 2 and 3). Of the total collected MPs, 80 plant species were used for the treatment of human ailments and 24 species were used against livestock diseases. Eleven (11) common plants species were listed in both which were used to treat both livestock and human ailments (Tables 2 and 3). The finding shows large numbers of MPs were used to treat human diseases compared with livestock ailments. This indicated that local people of the study area give much more priority of traditional medicine for their treatment as compared to their livestock ailments. As a result, they could acquire lower knowledge of MPs to treat livestock ailments than knowledge of MPs treating human ailments. Similar ethnobotanical findings were reported by Megersa et al. [23], Giday et al. [29], Getaneh [30], and Gebeyehu et al. [31].

The ethnobotanical study of MPs in Gozamin Wereda showed that plant medicines are used by a large amount of the population and it is the most important means of treating some common human and livestock ailments such as wound, stomachache, dysentery, diarrhea, gastritis, eczema, eye disease, snakebite, malaria, tapeworm, toothache, evil eye, cough, hemorrhoids, febrile illness, anthrax, control of leech, external animal parasites, tonsillitis, and fever especially for those community members who cannot buy the modern medicines with a relatively higher price.

#### 3.5.2. Diversity of Medicinal Plants in Natural Habitat and Home-Garden

**Figure 4:**
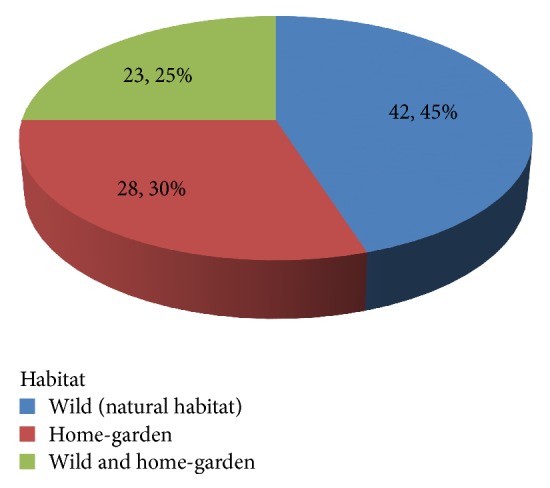
Diversity of medicinal plants in natural habitat and home-garden.

Most medicinal plants were collected in their natural habitat which account for 42 (45%) whereas 28 (30%) medicinal plants were from home-garden and 23 (25%) were from both home-garden and the wild (Figure 4). Various studies conducted in Ethiopia as well as other countries in the world reported that majority of MPs were harvested from the wild [32–34]. Here, our findings revealed that many MPs were also collected from home-gardens. This indicated that the people and the local healers have started cultivating many MPs in their gardens.

#### 3.5.3. Plant Parts Used in the Study Area

**Figure 5:**
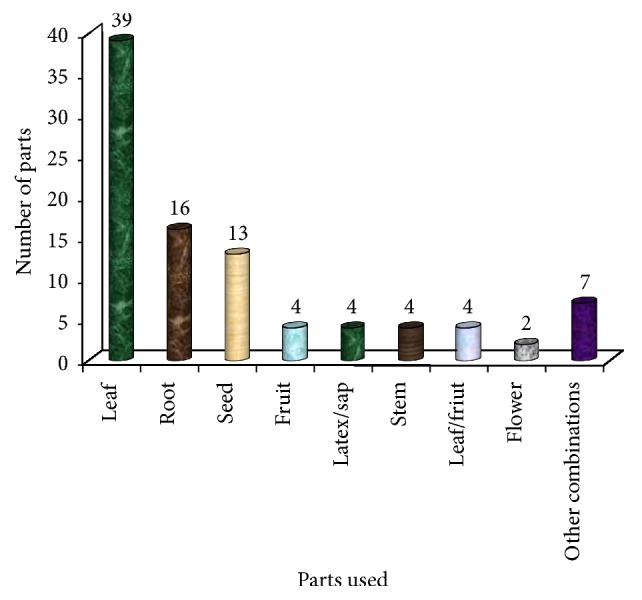
Plant parts used by Gozamin's community.

Every part of different plant species is used against a variety of ailments. As per the informant's response, the most commonly used part is the leaf (39), followed by root (16), seed (13), fruit, latex, leaf/or fruit, and stem (4, each) (Figure 5). In some cases, more than one organ of the same plant species, particularly a combination of parts, is used in the preparation of different therapies (see also Tables 2 and 3). It was also observed that the indigenous people have deep knowledge and age-old experience in the right mix of different plant parts to get better treatment of the given ailment. In this study, leaves are the most frequently utilized part of plant organs. Analogous results on different ethnobotanical studies by Giday et al. [12], Megersa et al. [23], Kefalew et al. [24], Gebeyehu et al. [31], Tolasa [32], and Lulekal et al. [33] were reported in Ethiopia.

It was reported that the ease of accessibility to leaves explains their frequent inclusion in most of the preparations [35]. It was also observed that residents have been using leaves to identify MPs. Additionally, leaves are the main photosynthetic organs in plants, and photosynthates are translocated to other parts, such as the root, stem, fruit, and seed. These can act as toxins for protection of predators and some are of medicinal value to humans.

On the other hand, the results of the study showed that harvesting of roots has great impact on the plants and leads to the dearth of the MPs. Fortunately, the plant parts which are mostly used for the preparation of the remedies in the study area were leaves and harvesting of leaves has less impact on the plant than harvesting of roots.

### 3.6. Disease Categories in the Study Area

**Figure 6:**
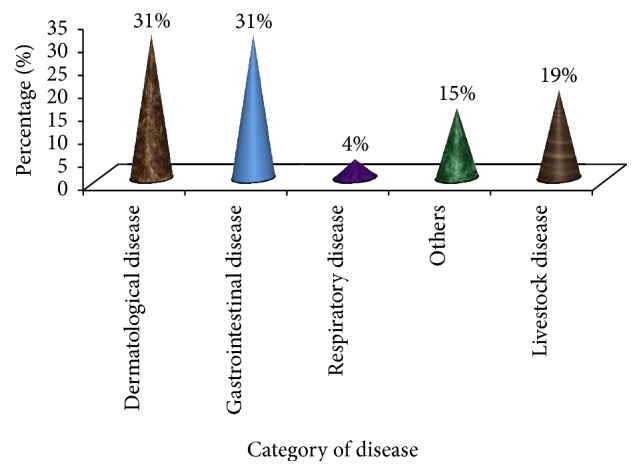
Categories of disease.

Diseases can be categorized in different ways as dermatological, respiratory, gastrointestinal, and so on. Based on the information obtained in the study site, the most prevalent diseases are related to skin (dermatology) and gastrointestinal ones (31%, each) followed by livestock (19%) and other diseases (15%) (Figure 6). The study showed that dermatological diseases such as wound, skin rush, itching, and eczema are prevalent and a number of MPs were found to be cited so as to treat these skin diseases. Similarly, gastrointestinal diseases like stomachache, gastritis, abdominal pain, intestinal parasite, dysentery, diarrhea, vomiting, hepatitis, and other related diseases were treated by many MPs. Respiratory and other diseases were also treated by various MPs (Figure 6; Tables 2 and 3).

The health problems could be as inverse as the MPs and associated ecological zones. For example, Tolasa [32] reported 49 human health problems, which have been treated by 85 different MPs. Awas and Demissew [36] and Gebeyehu et al. [31] identified skin disease as the most commonly reported health problems in Kafficho people, Menjarna Shenkora and Mecha districts, respectively. On one hand Belayneh et al. [37] and Lulekal et al. [27] reported gastrointestinal diseases as the major human health problems. On the other hand, Mesfin et al. [34] reported malaria as the most common human health problem of Wonago district.

### 3.7. Diversity of Medicinal Plants in terms of Families

**Figure 7:**
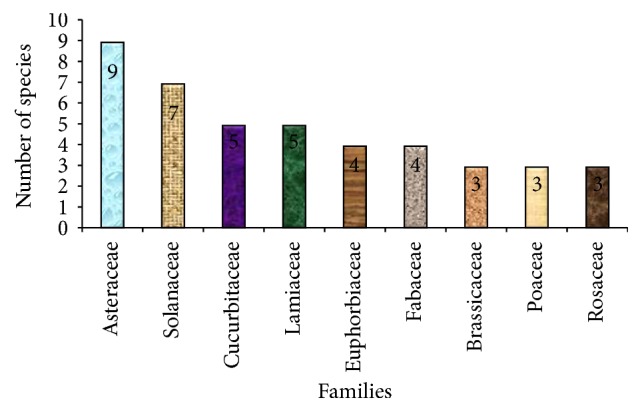
Families represented by highest number of medicinal plant species.

In this study different families of MPs were recorded. Among them Asteraceae is the most dominant family that holds 9 (9.68%) plant species followed by Solanaceae having 7 (7.53%) species. Other taxa commonly used are Cucurbitaceae and Lamiaceae (each 5 spp.), Euphorbiaceae and Fabaceae (each 4 spp.), Brassicaceae, Poaceae, and Rosaceae (each 3). The remaining forty-two (42) families hold fifty plant species. Eight of them account for two species. The rest of the families signify one species (Figure 7; Tables 2 and 3). Our finding agreed with the finding of Gebeyehu et al. [31] in which Asteraceae is the dominant family followed by Solanaceae. Etana [38] also reported that Asteraceae is the dominant family followed by Fabaceae. This may be because the weedy nature of the family Asteraceae takes advantage of disturbance.

### 3.8. Habits of Medicinal Plants Which Treat Diseases

**Figure 8:**
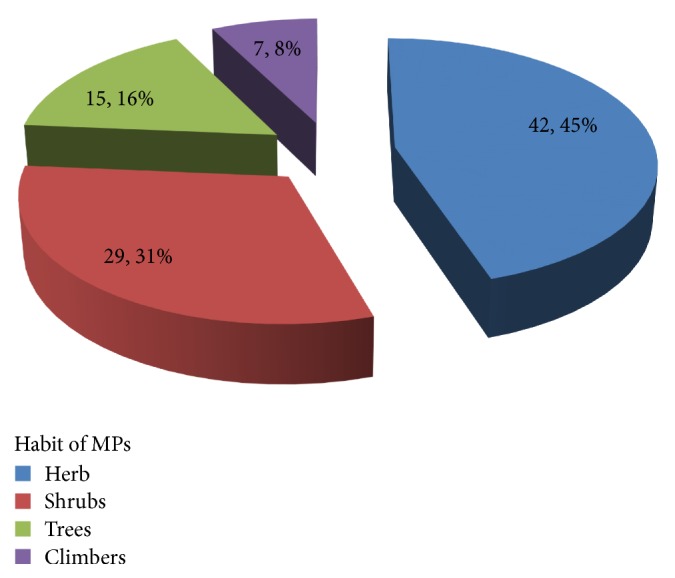
Habits of medicinal plants.

The finding shows that the most widely used MPs habits in the different Kebeles of the study areas were herbs, 42 (45%), followed by shrubs 29 (31%). Trees and climbers account for 15 (16%) and 7 (8%), respectively (see Figure 8). Herbs are largest in number; this may be because the plant species exhibit high level of abundance and it is easy to access them. The results of this finding agreed with the findings of other indigenous researchers including Berhan et al. [15], Giday et al. [12], and Teklehaymanot and Giday [39]. On the contrary, the findings of Hunde et al. [14], Giday and Ameni [9], Lulekal et al. [33], and Mesfin et al. [34] revealed that shrubs are the most commonly used habits in their respective different study sites of Ethiopia.

### 3.9. Traditional Methods of Preparation, Condition, and Route of Application

**Figure 9:**
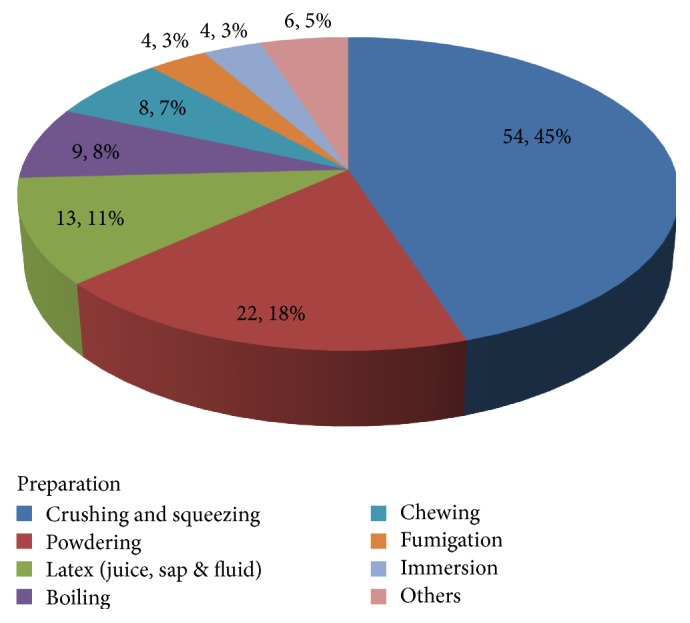
Ways of preparation of traditional medicine in the study area.

The popular method of preparation of traditional medicine is crushing and squeezing accounting for 54 (45%) followed by powdering (powder form), 22 (18%). It was also recorded that 13 (11%) were prepared in juice form, 9 (8%) in boiling form, 8 (7%) in chewing form, and 4 (3%) in the form of fumigation and immersion (each). Other methods such as paste form, raw form, and the like accounted for 6 (5%) (Figure 9). Methods can be used for human and livestock problem except chewing which is used only for humans.

Based on the informants' information the most popular method of preparation of remedy in the study area was crushing. This finding is in line with the results of Yineger and Yewhalaw [16], Getaneh [30], and Amsalu et al. [28]. However, Mesfin et al. [34], in a similar study on people of Wonago Wereda, reported that powdering was dominant method of preparation of remedy.

**Figure 10:**
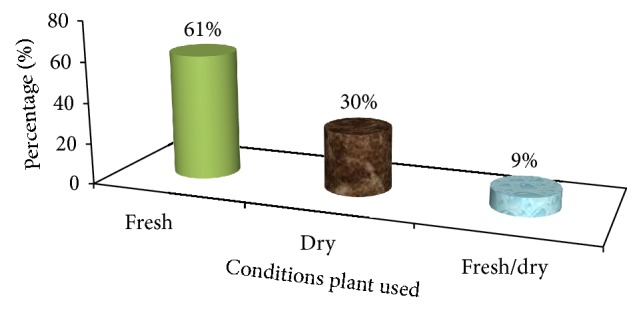
Conditions of preparations of traditional medicine in the study area.

The result in the conditions of plant part used indicated that most medicines (about 61%) of traditional MPs were prepared from fresh plant materials in the study site whereas 30% and 9% of medicinal plants were reported to be used dry and in both dry or fresh form, respectively (Figure 10). The most common use employs fresh (intact or pounded/crushed) leaves for external administration.

This finding is also consistent with the finding of [23, 26, 28–30, 32, 34, 36, 40] that reported that the majority of the remedy preparations were in fresh form. In contrast with this, Tolasa [32] stated that 60% of the preparations are fresh or dried followed by fresh, 36.47%, and dried, 14%.

Respondents argued that they use fresh plant parts mostly because they believe that using fresh materials increases efficacy as compared to the dry ones. This is because of the fact that the ingredients may be lost or reduced when the plants became dry. Nevertheless, this contributes much a lot to the threats of MPs given that local people have no practice of preserving dry form of traditional medicine.

**Figure 11:**
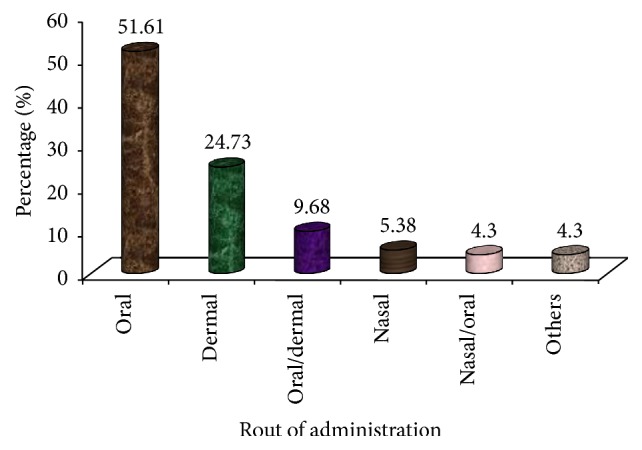
Route of administrations.

On the other hand, the most common route of administration is internal particularly oral that accounted for 51.61% followed by dermal, 24.73%. The oral/dermal, nasal, and nasal/oral ones and others are indicated in Figure 11. This result is being in agreement with the findings made by Megersa et al. [23] and Amsalu et al. [28], who reported that the leading route of application used by the given community in their respective study areas of remedies is taking orally.

#### 3.9.1. Dosage

Dosages were estimated using lid spoons, pinches, or handfuls (“woket”) (for powder preparations), cups (“sini” or “finjal”), “tassa” (can), and glasses (birchiko) (for liquid mixtures to be administered), numbers or in some cases handfuls (for leaf, seed, and fruits), and “atik” (for roots, stems, or barks). The measurements used to determine the dosages are not standardized and depend on the age, physical appearance of the patient, degree of the illness, diagnosis, and experience of individual herbalists/knowledgeable person. Children are given less than adults, such as one-fourth of a coffee cup whereas an adult is given up to one glass depending on the type of illness and treatment. Getahun [40] and Abebe [41] were also reporting independently lack of precision and standardization as drawback for the recognition of the traditional healthcare system. Giday and Ameni [9] also reported similar results.

### 3.10. Ranking of Medicinal Plants

#### 3.10.1. Preference Ranking

If a number of species are prescribed for the same ailment, people would tend to show preference of one over the other. Thus, preference ranking of six MPs which were reported against leach (livestock disease) was conducted after selecting six key informants. The informants were asked to compare the given MPs based on their efficacy and to give the highest number (5) for the medicinal plant which they thought most effective against leech and the lowest number (1) for the least effective plant in treating the disease. The results showed that* Nicotiana tabacum* was the most preferred followed by* Rhamnus prinoides* and* Solanum marginatum* (see Table 4).

Result of the preference ranking exercise also indicated that* Nicotiana tabacum* is the most preferred ethnoveterinary MPs used to treat leech, the most commonly reported disease in the area. This may be attributed to the presence of bioactive compounds against leech in this plant species. Hence, the species should be further investigated in the laboratory for further activities and even against different ailments.

#### 3.10.2. Paired Comparison

In this study, seven key informants made the pairwise comparisons of five MPs and the values were summarized as follows. It was found that* Acmella caulirhiza* species stood first followed by* Laggera tomentosa* for the treatment of toothache.* Olea europaea *subsp*. cuspidata, Solanum marginatum*, and* Momordica foetida* were placed 3rd, 4th, and 5th, respectively (Table 5). This rank is because of the effectiveness of the plant in the point of view of the indigenous people of Gozamin Wereda.

#### 3.10.3. Direct Matrix Ranking

Many MPs were found to be used for different purposes in addition to their medicinal values. The major uses include firewood, furniture, forage, charcoal, and eating. For ranking seven key informants were asked to give value, 5 to the most used plant for that particular purpose and 0 to the least used one. In view of that,* Cordia africana* was found to be the most multipurpose plant scoring 93, followed by* Carissa spinarum *scoring 87, and the least one was* Croton macrostachyus* having score of 72 (Table 6). The highest direct matrix ranking on the topic of* Cordia africana *was also reported in Gebeyehu et al. [31]. This confirms that as the value is getting high the plants have multiple uses in the context of the local community.

Direct matrix of randomly selected MPs with different uses including medicinal value on given use criteria revealed that MPs broadly collected for different purposes such as charcoal, construction, fencing, firewood, forage, furniture, and the like were also indicated [28, 38].

### 3.11. Jaccard's Coefficient of Similarity (JCS)

Jaccard's Coefficient of Similarity (JCS) revealed that the study area has the highest similarity with 45 common species (34.66%) to the study conducted around Wonago Wereda, followed by 40 common species (28%) with Debre Libanos Wereda, followed by 39 common species (21.91) with Chelya Wereda, followed by 30 common species (17.65) having similarity to Gimbi Wereda, followed by 23 common species (17.16%) having similarity to Zegie. The least similarity was linked with the study conducted on Bale Mountain National Park (Table 7).

### 3.12. Fidelity Level

Fidelity levels were calculated for* Plumbago zeylanica*,* Prunus africanus, Solanum anguivi *Lam., and* Withania somnifera* having highest scores (100%) which treat diseases like wounds, eczema, and fibril illness (Table 8). On the other hand,* Rhamnu*s* prinoides* which treats tonsillitis, skin infection, and dysentery has the least score (50%) (Table 8). Plants that are known as remedies of a single ailment have 100% fidelity level as compared to those that are used as remedies for more than one type of aliment. Most of the plants with high FL values have pharmacological effects that have been proven scientifically. On the other hand, the lowest one indicated less preferred species for treating specific ailments. In contrast, these plants have been widely used against several diseases. Plants with highest fidelity level values could also be targeted for further photochemical investigation to prove the bioactive components that are responsible for their high healing potential [42].

### 3.13. Nutraceuticals

Of the total MPs collected, twenty (21.51%) of them are used as a source of both medicine and food (nutraceuticals). The ethnobotanical information revealed that many food crops have medicinal effects (Table 9). The following plants like* Allium sativum, Brassica carinata*,* Brassica nigra*, and so on are directly used as a source of medicine and food in the study area of the given community. From this one can easily understand that the local people have IK and age-old experience in the use of plants as a source of medicine and nutrition. Similar findings were reported by Reta [43]. Most TMPs are safe, some are nutraceuticals, some are functional foods (wild fruits, vegetables, and other crops), and hence they are preferred.

### 3.14. Threatened and Endemic Medicinal Plants in the Study Area

In the study area, ethnobotanical information disclosed that MPs like* Hagenia abyssinica, Olea europaea *subsp.* cuspidata, Podocarpus falcatus, Juniperus procera, Euphorbia abyssinica, Myrica salicifolia, Cucumis ficifolius, Withania somnifera, Cordia africana, Brucea antidysenterica, Ficus sur, *and* Millettia ferruginea* are highly threatened. Medicinal plants documented as endemic from Ethiopia to the study site were* Brassica carinata, Eragrostis tef, Cynoglossum coeruleum, Echinops kebericho, Erythrina brucei, Guizotia abyssinica, Inula confertiflora, Lippia adoensis, Lobelia rhynchopetalum, Millettia ferruginea, Solanecio gigas*, and* Urtica simensis* which accounted for 13% (12 plant species) of the total collected MPs. Out of these,* Cynoglossum coeruleum, Inula confertiflora, Laggera tomentosa*, and* Lobelia rhynchopetalum* are nearly threatened while* Lippia adoensis, Solanecio gigas*, and* Urtica simensis* were under least concern (LN) [44]. This list could be used for collection of the rare plants of Ethiopia and contribute to national plant conservation target.

### 3.15. Threats and Conservation of Medicinal Plants in the Study Area

#### 3.15.1. Threats of Medicinal Plants

As elsewhere in Ethiopia, plant resources are vital for the livelihood of the Amhara people of Gozamin community. In the study sites, the resources are eroded from time to time because of the increment of population. Associated with this, the demand of agriculture (raring of livestock and cultivation) is high and therefore overgrazing and clearance of vegetation/forests are high. There is evidence of remnant plants at the spot in the grazing lands and farmlands of representative Kebeles in the Wereda where the data were collected. This indicates that overgrazing and deforestation were the main cause of the devastation of plants in the study area. Priority ranking factors (Table 10) also indicated that overgrazing contributed the major factor (26.12%) to the threat of MPs followed by charcoal and firewood (23.42%) and deforestation (23.42%) (for agricultural expansion, furniture, and building). Drought and mining are other destructive factors, which account for 14.41% and 12.61% of the total scores, respectively.

Almost all of the informants are familiar with one and more than one threat for the scarcity of medicinal plants in the study area. Deforestation for firewood, charcoal, construction, agriculture, and mining is common practice in the study areas. The previous vegetation site has been changed drastically and the most useful plant species are at risk and they are on the way to be vanished. Similarly, Balemi et al. [13] reported that overgrazing was principal threat to MPs in Fentalle area. On the other hand, the findings of Leulekal et al. [33], Megersa et al. [23], and Chekole et al. [25] indicated that intense deforestation became the major threat on MPs in their respective study sites. Berhanu et al. [15] and Kefalew et al. [24] also reported that agricultural expansion was the major factor contributing to the local decline of medicinal plants.

### 3.16. Conservation and Management Practices

In Gozamin Wereda, irregular remnants of aged dry Afromontane evergreen forests that contain many MPs can be found mainly around the Ethiopian Orthodox Tewahedo Churches. Hence, someone sees a patch of indigenous old-aged trees in the study area; he/she can be sure that there is an Orthodox Church in the middle. Patches of forests are visible from a great distance. Similar findings were reported in Chekole et al. [25]. This shows that culture, belief, and religion contribute much a lot to the conservation of MPs.

Some traditional practitioners in the study area have brought the different curative plans from different corners and started to conserve these MPs by cultivating at home-gardens. On the topic of this, Asfaw [11] in his findings reported that the home-garden is a strategic and ideal farming system for conservation, production, and enhancement of MPs and valuable IK. The same information was also documented by Etana [38].

Likewise, few management practices are carried out in the home-gardens of Gozamin's indigenous people. One of the practices is to the make an effort for maintaining diverse plant species in the garden as much as possible. Diversity is achieved through planting and protecting annual and perennial herbs and woody perennials in combinations. Management practices like intercropping and crop rotation were observed among very few farmers of the study area. In doing so, herbal remedies continued to exist because of the existence of other plant species. As a result, indigenous people can be excellent conservators of plant diversity. However, according to the informants, in most cases attempts regarding conservation were weak. Similarly, Lulekal et al. [27] reported that although traditional practitioners and local communities in their study sites mainly depend on the natural environment for collecting MPs, the effort to conserve and sustainably utilize resources was frail.

## 4. Conclusions

Through the ethnobotanical survey conducted from January 5, 2014, to February 15, 2015, a total of 93 MPs under 51 families and 87 genera were recorded and documented from 12 sample Kebeles of the local people for the treatment of different human and livestock ailments. The majority of the reported medicinal plant species were harvested from natural habitats. Furthermore, about 13% of medicinal plants of the study area were found endemic to Ethiopia. Both human and livestock health problems were most frequently treated by fresh plant material. Herbs were reported as the most dominant growth forms in the preparation of traditional remedies followed by shrubs, trees, and climbers. Leaves followed by roots were the dominant plant parts used for preparation of most remedies.

This study showed that traditional herbal medicine is playing a significant role in meeting the primary healthcare needs of Gozamin community. Acceptance of traditional herbal medicine and limited access to modern healthcare services could be considered as the main factors for the continuation of the practice. Some plant species were also reported to have uses other than their medicinal values. However, the efforts on the use and conservation of medicinal plants and associated indigenous knowledge were observed to be poor. Hence, awareness about the need for in situ and ex situ conservation should be created among the local communities and urgent measures must be given to threatened plant species.

## Figures and Tables

**Table 1:** Sociodemography of the informants.

Informants	Categories	Number	%
Sex	Male	88	88.00
	Female	12	12.00
	Total	100	100

Marital status			
Married	Male	56	56.00
	Female	10	10.00
	Total	66	66.00
Unmarried	Male	28	28.00
	Female	2	02.00
	Total	30	30.00
Divorced	Male	3	03.00
	Female	1	01.00
	Total	4	04.00
Grand total		100	100

Educational level			
Modern education		64	64.00
Church education		17	17.00
Uneducated/illiterate		19	19.00
Total		100	100

**Table 2:** List of medicinal plants used for treating human ailments in the study area with scientific name, family, local name, habit (Ha), shrub (S), tree (T), herb (H), climber (Cl), seed (Se), fruit (Fr), flower (Fl), shoot (Sh), collection number (Coll. No.) source, condition of preparation (fresh/F and dry/D), parts used (PU), root (R), leaf (L), latex (La), stem (St), bulb (Bu), bark (Ba), and rhizome (Rh).

Scientific name, family	Local name (Amharic)	Ha	Disease treated	PU	Preparations, conditions of plants used, and mode of applications	Route	Voucher number
*Achyranthes aspera* Lam. (Amaranthaceae)^*∗∗*^	Telenj	H	Hemorrhoids	R	Fresh roots pounded and applied on the infected part.	Anal	NA10

*Acmella caulirhiza* Del. (Asteraceae)	Yemidir berbere	H	Tonsillitis Toothache	Fl	Fluid extract from the leaf is drunk (one spoon). Flower is chewed for few minutes and spit out.	Oral	NA2

*Allium sativum *L. (Alliaceae)	Shinkurt	H	Cough	Bu	Its bulb and fruit of *Capsicum annum *areboiled with butter and drunk continuously.	Oral	NA7

*Aloe macrocarpa* Tod. (Alliaceae)	Ret	H	Stomachache	La	The latex of the species is squeezed and taken once.	Oral	NA20

*Arisaema schimperianum* Schott. (Araceae)	Amoch	H	Birth difficulty	R	Fingertip size fresh root is squished and mixed with alcohol (local arki), and then one cup is drunk.	Oral	NA80

*Artemisia afra* Jacq. ex Wilk (Asteraceae)	Chiqugn	H	Evil eye Malaria	L	Fresh leaf and *Allium sativum *bulb squeezed and wrapped together in a piece of cloth and sniffed through nose. Fresh leaf crushed and pounded with water and then filtered and drunk in one tea cup.	Nasal Oral	NA75

*Asparagus africanus* Lam. (Asparagaceae)	Yeset qest	S	Impotency	R	The root powder along with *Premna schimperi* is given with one cup of local alcohol (tella).	Oral	NA9

*Brassica carinata *A. Br. (Brassicaceae)^**∗**^	Gomen	H	Skin rash	Se	The powder is mixed with water and applied on the skin.	Dermal	NA60

*Brassica nigra *(L.) Koch (Brassicaceae)	Sinafich	H	Abdominal pain	Se	Powder of dried seeds with seeds* of Lepidium sativum *mixed with water and one cup is drunk.	Oral	NA45

*Brucea antidysenterica* Swiss Chard. (Simaroubaceae)	Avalo	T	Bloody diarrhea Wound Leishmaniasis	R/L Fr Se	Dry root is crushed and dissolved in water or the leaf is squeezed and 1/2 of coffee cup is drunk. Dried fruit finely crushed and applied on wounds. Dried seed crushed, added with wheat flour, and then applied on wounds.	Oral Dermal Dermal	NA1

*Buddleja polystachya* Fresen. (Loganiaceae)	Anfar	S	Wound	L	Dried leaf finely crushed and mixed with *Lepidium sativum* and applied on wounds.	Dermal	NA22

*Calpurnia aurea* (Ait.) Benth. (Fabaceae)	Ligita	S	Hepatitis Wound	L	The leaves are boiled with a cup of water and then drunk for 6–10 days. Fresh leaves pounded and applied on the wounds.	Dermal/oral Oral	NA90

*Capparis tomentosa* Lam. (Capparidaceae)^*∗∗*^	Gumero	S	Evil sprit	R	Dry root crushed and boiled with water and then fumigated or inhaled.	Nasal/oral	NA6

*Capsicum annuum *L. (Solanaceae)	Qaria	H	Dysentery, vomiting	Fr	Its fruits with *Allium sativum, Zingiber officinale, *and *Nigella sativum* are immersed in water for 2-3 days and drunk continuously.	Oral	NA28

*Carduus schimperi* Sch. Bip. ex A. Rich. (Asteraceae)	Kosheshila	H	Fibril illness	R	The root is pounded, squeezed, and drunk.	Oral	NA30

*Carica papaya* L. (Cucurbitaceae)	Papya	Cl	Gastritis Malaria Diarrhea	Se L Se	The seed can be eaten together with the fleshy part. Freshly crushed, boiled, and drunk. Ingest a few seeds with “Injera” for three days.	Oral	NA17

*Carissa spinarum* L. (Apocynaceae)	Agam	S	Evil eye	R	Dried root bark powder is added on fire and allowed to be inhaled.	Nasal/oral	NA51

*Clutia abyssinica* Jaub. and Spach (Euphorbiaceae)	Fiyele-fej	S	Dysentery	St	Crush fresh stem making it powder and then drink.	Oral	NA59

*Coffea arabica* L. (Rubiaceae)	Bunna	S	Wound	Se	Seeds roasted, powdered, and put on the infected skin.	Dermal	NA3

*Cordia africana* Lam. (Boraginaceae)	Wanza	T	Vomiting	Fl	Flowers crushed or smashed and swallowed.	Oral	NA4

*Croton macrostachyus* Del. (Euphorbiaceae)	Bisana	T	Stomachache Ringworm Malaria	L La L	Squeezing the fresh leaf and drinking the droplet. Sap applied topically on the skin. Boil fresh leaf in water, filter, and drink with milk or tea.	Oral Dermal Oral	NA12

Cucumis ficifolius A. Rich (Cucurbitaceae)	Yemidir Embuy	Cl	Cough	R	Fresh roots are washed, smashed, and mixed with water overnight. Then, mix the suspensions with honey and milk and drink one glass.	Oral	NA46

*Cynoglossum coeruleum* (Hochst. A. Rich.) DC (Boraginaceae)^**∗**^	Shimgug	H	Stomachache Sun-strike	R L	The root is washed and cleaned, and then the liquid is chewed and taken. Leaf is squeezed and applied on the entire body.	Oral Dermal	NA56

*Dodonaea angustifolia* L. f. (Sapindaceae)	Kitkita	S	Sore/eczema	L	Dry leaves along the leaves of *Citrus lemon *arepowdered and mixed with butter and then applied on the affected part.	Dermal	NA63

*Datura stramonium* L. (Solanaceae)	Astenagir	H	Dandruff	L	Fresh leaves pounded and smashed with hand and then applied on the head like ointment.	Dermal	NA61

*Dracaena steudneri* Engl (Dracaenaceae)^*∗∗*^	Merqo	T	Emergency (“dingetegna”)	R	The root is crushed and mixed with water; one cup is given to drink.	Oral	NA67

*Echinops kebericho* Mesfin (Asteraceae)^**∗**^	Qebercho	H	Scabies	R	The root is powdered and applied on the affected area at bedtime.	Dermal	NA69

*Embelia schimperi* Vatke (Myrsinaceae)^*∗∗*^	Enqoqo	S	Tapeworm	Fr	Dried fruit is powered and dissolved by water and then one glass water is drunk.	Oral	NA73

*Eragrostis tef* (Zucc.) Trotter (Poaceae)^**∗**^	Dawa tef	H	Bone fracture	Se	Flour of teff is mixed with different spices and then eaten continuously.	Oral	NA83

*Erica arborea* L. (Ericaceae)	Asta	S	Circumcision	L	Fresh leaf is mixed with leaves of *Verbascum sinaiticum* and pounded together and then powder is put on affected part.	Dermal	NA5

*Erythrina brucei *Schweinf. (Fabaceae)^**∗**^	Korch	T	Eczema	L/ba/R	The powders of dried leaf, bark, and root are mixed with cheese and then applied.	Dermal	NA25

*Eucalyptus globulus *Labill. (Myrtaceae)	Nech bahir zaf	T	Asthma Athlete's foot	L L/Sh	The young leaf is boiled with water and the steam is inhaled. Washing the sol with young shoot or putting under the sock.	Nasal/oral Dermal	NA87

*Euphorbia abyssinica* Gmel (Euphorbiaceae)^*∗∗*^	Qulqual	T	Jaundice, rabies	Sap	Few droplets of the milky latex are mixed with the powder of wheat and teff and then baked and eaten.	Oral	NA91

*Ficus sur *Forssk. (Moraceae); NA55	Sholla	T	Dysentery	La	About one spoon of sap is taken.	Oral	NA55

*Foeniculum vulgare* Miller (Apiaceae)	Ensilal	H	Abdominal pain	Se	The leaves boiled with tea or coffee and then drunk.	Oral	NA35

*Hagenia abyssinica* (Bruce) J.F.Gmel (Rosaceae)	Kosso	T	Tapeworm	Se	Dry seed ground into powder, mixed with local alcohol (“tella”), and drunk.	Oral	NA37

*Hordeum vulgare* L. (Poaceae)	Gebis	H	Gastritis	Se	The roasted powder is boiled in water and drunk until relief.	Oral	NA39

*Jatropha curcas* L. (Euphorbiaceae)	Jatrofa	T	Wound	La	The latex is applied on the wound.	Dermal	NA78

*Jasminum abyssinicum L.* (Oleaceae)	Tenbelel	Cl	Tapeworm	L	Fresh leaves crushed, squeezed mixed with water, and decanted and then drunk without having food.	Oral	NA14

*Justicia schimperiana *(Hochst. ex Nees) T. Anders (Acanthaceae)^**∗∗**^	Smiza	S	Wound Abdominal parasite	L	The fresh leaf paste is applied over infected area of skin. Dry leaves are crushed and pounded with water and then one glass is drunk.	Dermal Oral	NA15

*Kalanchoe petitiana* A. Rich. (Crassulaceae)	Andahula	H	Swelling Tonsil	L/St R	Fresh leaves or stem heated with fire and put on the swollen part. Fresh root is put on the nostril.	Dermal Nostril	NA53

*Laggera integrifolia* Sch. Bip. ex A. Rich (Asteraceae)^**∗**^	Gimmie	H	Common cold Toothache	L	Leaf is inhaled for sometimes through nose. Crushed and put b/n teeth for 5–10 minutes and then spit out.	Nostril Mouth	NA8

*Leonotis ocymifolia* (Burm F.) A. Iwarsson. (Lamiaceae)	Feres zeng	H	Diarrhea	Fr/L	Powder of dried fruit and leaf is mixed with honey and then given.	Oral	NA23

*Linum usitatissimum* L. (Linaceae)	Telba	H	Gastritis	Se	Few seeds immersed in water over night and one glass drunk continuously.	Oral	NA24

*Lepidium sativum* L.(Brassicaceae)	Feto	H	Diarrhea	Se	Seeds ground into paste-like food and then eaten or mixed with butter and water and drunk.	Oral	NA74

*Lippia adoensis* Hochst ex. Walp. (Verbenaceae)^**∗**^	Kesiy	H	Gastritis	L	Fresh leaves chewed.	Oral	NA26

*Lobelia rhynchopetalum* (Hochst) Hemsl. (Campanulaceae)^**∗**^	Jebera	T	Evil sprit	R	The dried root fumigated.	Nasal/oral	NA40

*Lupinus albus* L. (Fabaceae)	Gibto	H	Hypertension	Se	Soaking with water for 3–5 days, decanting the water, and eating and/or preparing in the form of alcohol and drunk.	Oral	NA72

*Maytenus arbutifolia* (A. Rich.) Wilczek (Celastraceae)	Atat	H	Itching	R	Dry root is boiled in water and body is washed with it	Dermal	NA76

*Millettia ferruginea* (Hochst.) Bak. (Fabaceae)^**∗**^	Birbira	T	Skin infection Goiter	Fr	Dry fruit powder is mixed with butter and salt and then applied to the infected skin. Chewing the fruit for goiter.	Dermal Oral	NA77

*Momordica foetida* Schumach. (Cucurbitaceae); NA33	Yamora misa	Cl	Toothache Wound	L	Fresh leaf is chewed for sometimes and spit out. Fresh leaf is crushed and pasted.	Oral Dermal	NA33

*Myrica salicifolia* A. Rich. (Myricaceae)	Shinet	T	Leishmaniasis	Ba	The powder is mixed with butter and applied on the contaminated part.	Dermal	NA32

*Myrtus communis* L. (Myrtaceae)	Ades	S	Ringworm	L	Leaf powder is mixed with butter and is being applied on the infected part continuously.	Dermal	NA31

*Nigella sativa* L.(Apiaceae)	Tiqur-azmud	H	Common cold Asthma	Se Se	The seeds ground into powder and covered with a piece of cloth and inhaled three to four times per day. Seeds are put in boiling water and steam is inhaled.	Nasal Nasal/oral	NA34

*Ocimum gratissimum* L. (Lamiaceae)	Ziqaqibie	H	Dysentery	L	Leaf is pounded, mixed with water, and drunk.	Oral	NA49

*Ocimum lamiifolium* Hochst. ex Benth. (Lamiaceae)	Dama kesy	H	Sun-strike	L	From three places the leaves are cut and crushed, squeezed, and drunk.	Oral	NA48

*Olea europaea* L. subsp*. cuspidate* (Wall. Ex G. Don) Cif. (Oleaceae)	Woira	T	Toothache	St	Dry/fresh branches used as tooth brush.	Oral	NA68

*Otostegia integrifolia* Benth. (Lamiaceae)	Tinjut	S	Abdominal pain	L	Pounding and squeezing the fresh leaf and mixing with water then drinking.	Oral	NA47

*Phytolacca dodecandra* L'Herit. (Phytolaccaceae)	Endod	S	Abortion	L	The leaf is crushed, mixed with water, filtered, and drunk.	Oral	NA11

*Plantago lanceolata* L. (Plantaginaceae)	Gorteb	H	Cut	L	Crush and apply it on the cut part.	Dermal	NA13

*Plumbago zeylanica* L. (Plumbaginaceae)	Amera	H	Wounds	R	Dried root finely crushed and applied or rubbed on wounds.	Dermal	NA18

*Podocarpus falcatus* (Thunb.) R. B. ex. Mirb. (Podocarpaceae)	Zigba	T	Swelling	L	Fresh leaf is crushed and tied with a piece of cloth on the contaminated skin.	Dermal	NA19

*Prunus africana* (Hook. F.) Kalkman (Rosaceae)	Homma	T	Wound	Ba	Powdered and tied for 5–7 days.	Dermal	NA16

*Ranunculus oligocarpus* Hochst. ex A. Rich. (Ranunculaceae)	Etsesiol	H	Eczema	L	Crushing and tying the infected part with a piece of cloth for two hours.	Dermal	NA29

*Rhamnus prinoides* L'Herit (Rhamnaceae)^**∗∗**^	Gesho	S	Tonsillitis Skin infection Dysentery	Fr L L	The dry fruit of the plant is made as powder and mixed with water and then drunk. It is rubbed with the fresh leaves on the infected skin as gel. Fresh leaves are squashed and mixed with water, and about one cup is drunk.	Oral Dermal Oral	NA27

*Rumex nepalensis* Spreng (Polygonaceae)	Tult	H	Blood pressure Stomachache	R	About the size of fingertip of root is chewed and the juice is taken daily. Chewing and taking the sap.	Oral Oral	NA38

*Rumex nervosus* Vahl (Polygonaceae)	Enbuacho	S	Burn	St	The powder of roasted stem is mixed with butter and applied on the skin.	Dermal	NA43

*Ruta chalepensis* L. (Rutaceae)	Tila-adam	H	Common cold Stomachache	St and L L	Fresh stem and leaf are boiled with coffee or tea then drunk. The fresh leaf is immersed with cold water for 24 hrs and drunk.	Oral Oral	NA42

*Sida schimperiana* Hochst. ex A. Rich. (Malvaceae)	Chifrg	S	Dysentery	L	The dried leaf is ground and mixed in a cup of water. Drink two spoon solutions after shaking.	Oral	NA50

*Solanum anguivi *Lam. Hochst ex A. Rich. (Solanaceae)	Zercho enbuay	S	Eczema	Fr	The fruits along with *Myrtus communis *and spices are crushed, mixed with butter, and stained for consecutive days.	Dermal	NA41

*Solanum marginatum* L.f. (Solanaceae)^**∗∗**^	Enbuay	S	Toothache	Fr	The fruit sap is applied on affected tooth drop by drop.	Oral	NA44

*Solanum nigrum* L. (Solanaceae)	Awit	H	Itching	Fr	Watery/fluid of the fruit is applied on the skin part.	Dermal	NA85

*Stephania abyssinica* (Dill. & A. Rich.) Walp. (Menispermaceae)	Yayit jero	Cl	To increase mental activity	R	Dry smashed root is mixed with “Abish” and then drunk with one cup of coffee.	Oral	NA86

*Urtica simensis* Stedel (Urticaceae)^**∗**^	Samma	H	Gastritis	L Sh	Prepare in the form of stew and eat with bread (“Injera”)	Oral	NA88

*Verbasicum siniaticum* Benth. (Sclrophularaceae)^**∗∗**^	Qetentina	H	Gastritis	R	Juice is extracted from fresh root and then drunk.	Oral	NA70

*Verbena officinalis* L. (Verbenaceae)	Atuch	H	Abdominal pain, diarrhea, stomachache Heartburn	R	Sap of the fresh root is chewed and swallowed for three days. Root is crushed, squeezed, and given to the patient.	Oral	NA71

*Vernonia amygdalina* Del. (Asteraceae)	Girawa	S	Stomachache Intestinal parasite	L	Fresh leaf is mixed with water, crushed, and squeezed, decanted, and drunk. Juice is extracted from fresh leaf and taken orally (one cup).	Oral	NA92

*Withania somnifera* (L.) Dunal (Solanaceae)	Gizewa	S	Fibril illness	L	Its leaf is crushed along with *Allium sativum *and the whole body is rubbed or the leaf is boiled with water and the vapour is inhaled.	Dermal	NA93

*Zehneria scabra* (Linn. F.) Sondll. (Cucurbitaceae)	Harg ressa	Cl	Sun-strike	L	It is crushed, mixed with water, and decanted, and then one cup is drunk or the stem is boiled and inhaled.	Oral	NA64

*Zingiber officinale *Roscoe (Zingibraceae)^**∗∗**^	Zinjible	H	Stomachache	Rh	Chewing and eating.	Oral	NA66

*Note*. ^*∗*^Endemic plant species. ^*∗∗*^Medicinal plant species which are used in the treatment of both human and livestock ailments.

**Table 3:** List of medicinal plants used for treating livestock diseases in the study area, with scientific name, family, local name, habit (Ha), shrub (S), tree (T), herb (H), climber (Cl), seed (Se), fruit (Fr), flower (Fl), shoot (Sh), collection number (Coll. No.), source, condition of preparation (fresh/F and dry/D), parts used (PU), root (R), leaf (L), stem (St), bark (Ba), and rhizome (Rh).

Scientific name, family	Local name (Amharic)	Ha	Disease treated	PU	Preparations, conditions of plants used, and mode of applications	Route	Voucher number
*Achyranthes aspera* Lam. (Amaranthaceae)^*∗∗*^	Telenj	H	Eye infection	R	Fresh root is crushed and mixed with water and then dropped into the cattle's eye.	Eye	NA10

*Capparis tomentosa* Lam. (Capparidaceae)^*∗∗*^	Gumero	S	Evil sprit	R	Fumigation of dried root.	Nasal/oral	NA6

*Clausena anisata (Willd.)* J. D. Hook. Ex. Benth. (Rutaceae)	Limich	S	Coccidiosis	L	The leaves are squashed and extracted by water then the juice is given to chicken pox one or two spoon with bread.	Oral	NA81

*Cymbopogon citratus *(DC.) Stapf. (Poaceae)	Tejesar	H	Anthrax/unspecified disease	R	Dried root powder is mixed with fresh water and then given to cattle.	Oral	NA82

*Dracaena steudneri* Engl. (Dracaenaceae)^*∗∗*^	Merqo	T	Evil sprit	Ba	The juice is extracted from inner part of the bark and mixed with water and then given to animals for 2-3 days.	Oral	NA67

*Embelia schimperi* Vatke (Myrsinaceae)^*∗∗*^	Enqoqo	S	Unspecified disease	L	Its fresh leaves along with the leaves of *Phytolacca dodecandra* could be crushed, squeezed, decanted and given to cattle.	Oral	NA73

*Euclea racemosa* (DC) Dandy (Ebenaceae)	Dedeho	S	Wound	L	Leaf powder is applied topically on the wounds of livestock.	Dermal	NA79

*Euphorbia abyssinica* Gmel. (Euphorbiaceae)^*∗∗*^	Qulqual	T	Rabies	R	The root crushed and mixed with food and then given to dog.	Oral	NA91

*Grewia ferruginea* Hochst. ex A. Rich. (Tiliaceae)	Lenquata	S	Placenta retention	L	Leaves pounded, mixed with water, and then given to cattle to drink.	Oral	NA21

*Guizotia abyssinica* (L.f.) Cass. (Asteraceae)^*∗*^	Nug	H	Leech	Se	The seed is pounded and boiled with water then after cooling applied through nasal cavity to expel the parasite one cup.	Nasal	NA84

*Inula confertiflora* A. Rich. (Asteraceae)^*∗*^	Woynagift	S	Infected eye	L	Fresh leaves crushed, squeezed, and dropped into eye.	Eye	NA89

*Justicia schimperiana* (Hochst. ex Nees) T. Anders. (Acanthaceae)^*∗∗*^	Smiza	S	Coccidiosis Unspecified disease (“Qurba”)	L	The leaves are squashed and extracted with water, then one or two spoons of juice are given to chicken. Its leaves along with the leaves of *Ricinus communis* are crushed, squeezed, mixed, and decanted, and then about one can is given to cattle.	Oral Oral	NA15

*Lagenaria abyssinica* (Hook. f.) C. Jeffrey (Cucurbitaceae)	Qil	Cl	Leech	Fr	Fresh fruit sap is given to cattle.	Nasal	NA54

*Melia azedarach* L. (Meliaceae)	Nim	T	Insect repellent	L	Leaves and juice sprayed in the house (on infected cattle skin).	Dermal	NA52

*Nicotiana tabacum* L. (Solanaceae)	Tinbaho	S	Leech	L	Leaves crushed, squeezed, and dropped into the cattle's nostril.	Nasal	NA62

*Otostegia integrifolia* Benth. (Lamiaceae)	Tunjit	S	Leech	L	Leaf juice is squashed and applied through nasal cavity to expel the parasite.	Nasal	NA47

*Prunus persica* (L.) Batsch (Rosaceae)	Kok	S	Diarrhea	L	Crushed and immersed in water for few minutes and then given to calf.	Oral	NA16

*Rhamnus prinoides* L' Herit (Rhamnaceae)^*∗∗*^	Gesho	S	Leech	L	Leaf juice is pounded with water and applied through nasal cavity to expel the parasite	Nasal	NA27

*Rubia cordifolia* L. (Rubiaceae)		H	Cough	Wh	Whole plant concoction is drunk (given to cattle).	Oral	NA65

*Salvia schimperi *Benth. (Lamiaceae)	Yahiya Jero	H	Coccidiosis	L	Fresh leaf is mixed with *Ruta chalepensis* leaf and *Allium sativum* bulb; squeezed juice mixed with water is given orally to hen.	Oral	NA58

*Solanecio gigas* Vatke. (Asteraceae)^*∗*^	Yeshikoko gomen	S	Dysentery Bloating	L	The whole leaf is given to cattle. The leaf is pounded, squeezed, and added through nose.	Oral Nasal	NA57

*Solanum marginatum* L.f. (Solanaceae)^*∗∗*^	Enbuay	S	Leech External parasite	Fr	The fruits' sap is dropped into nostril. The cattle's skin is rubbed with the fluid.	Nasal Dermal	NA44

*Verbasicum sinaiticum* Benth. (Scrophulariaceae)^*∗∗*^	Qetentina	H	Rabies	L	Juice squashed from fresh leaf and then given to the dog with milk and food.	Oral	NA70

*Zingiber officinale* Roscoe (Zingiberaceae)^*∗∗*^	Zinjibl	H	Stomachache	R	Fresh rhizome crushed is mixed with salt and then dissolved by water and one cup is given.	Oral	NA66

*Note*. ^*∗*^Endemic plant species. ^*∗∗*^Medicinal plant species which are used in the treatment of both human and livestock ailments.

**Table 4:** Preference ranking of six medicinal plants against leech in livestock (cited by six respondents).

Medicinal plants	Respondents (R_1_–R_6_)						Total	Rank
	R_1_	R_2_	R_3_	R_4_	R_5_	R_6_		
*Guizotia abyssinica*	3	2	2	2	2	1	12	6th
*Lagenaria abyssinica*	3	2	2	3	2	2	14	5th
*Otostegia integrifolia*	1	4	3	3	3	2	16	4th
*Rhamnus prinoides*	4	4	3	4	4	4	23	2nd
*Solanum marginatum*	4	4	3	4	4	4	23	3rd
*Nicotiana tabacum*	4	4	3	4	5	5	25	1st

*Note*. Scores in the table indicate ranks given to medicinal plants based on their efficacy. Highest number (5) is given to the medicinal plant which informants thought to be most effective in treating leech and the lowest number (1) is given to the least effective plant.

**Table 5:** Paired comparison of five medicinal plants used to treat toothache.

Medicinal plants	Respondents (R_1_–R_7_)							Total	Rank
	R_1_	R_2_	R_3_	R_4_	R_5_	R_6_	R_7_		
*Acmella caulirhiza*	3	4	4	4	3	5	3	27	1st
*Laggera tomentosa*	3	4	3	3	3	3	4	23	2nd
*Olea europaea *subsp.* cuspidata*	2	3	3	3	3	2	3	19	3rd
*Solanum marginatum*	3	2	2	3	2	2	3	17	4th
*Momordica foetida*	2	2	2	2	3	2	3	16	5th

*Note*. A paired comparison of five highly cited MPs used to treat a highly cited human ailment (toothache).

**Table 6:** Direct matrix ranking for the multipurpose of six medicinal plants [average score of 7 key informants (5 to the most used plant for that particular purpose and 0 to the least used one)].

Medicinal plants	Use categories						Total	Rank
	Medicine	Firewood	Furniture	Forage	Soil con.	Edible		
*Cordia africana*	24	20	24	5	10	10	93	1st
*Carissa spinarum*	27	17	5	10	12	16	87	2nd
*Prunus africanus*	20	21	20	5	8	8	82	3rd
*Olea europaea *subsp*. cuspidate*	20	22	14	5	9	5	75	4th
*Vernonia amygdalina*	30	20	15	0	8	0	73	5th
*Croton macrostachyus*	29	19	18	0	6	0	72	6th

**Table 7:** Jaccard's Coefficient of Similarity index with six other areas with respect to plants species composition.

Sample of study areas	A	B	C	JCS%	References
Gozamin Wereda	93	-	-	-	Present
Bale Mountain National Park	101	86	15	7.42	Yineger et al. 2008
Chelya District	89	50	39	21.91	Amenu, 2007
Debre Libanos District	90	50	40	22.22	Getaneh, 2009
Gimbi District	85	55	30	17.65	Tolasa, 2007
Wonago District	65	20	45	34.66	Mesfin et al. 2009
Zegie Peninsula	67	44	23	17.16	Teklehaymanot and Giday, 2007

**Table 8:** Fidelity level of traditional medicinal plants cited by informants against the corresponding human ailment.

Plant species	Diseases treated	*NP*	*N*	FL values (%)
*Plumbago zeylanica*	Wounds	7	7	100
*Prunus africanus *	Wounds	8	8	100
*Solanum anguivi *Lam.	Eczema	10	10	100
*Withania somnifera*	Fibril illness	5	5	100
*Verbena officinalis*	Stomachache and intestinal parasite	12	15	80
*Rumex nepalensis *	Blood pressure and stomachache	18	24	75
*Brucea antidysenterica *	Diarrhea, wound and leishmaniasis	15	20	75
*Laggera tomentosa*	Common cold and toothache	25	35	71.42
*Carica papaya*	Gastritis, malaria, and diarrhea	25	45	55.56
*Rhamnus prinoides*	Tonsillitis, skin infection, and dysentery	20	40	50

FL = fidelity level; *NP* = number of informants who independently cited the importance of a species for treating a particular disease; *N* = total number of informants who reported the plant for any given disease.

**Table 9:** Lists of nutraceutical (used as both food and medicine) plants.

Botanical names	Family	Local names (Amharic)	Uses as food	Disease treated
*Allium sativum *	Alliaceae	Nech shinkurt	Bulb and leaf as food	Cough, evil eye, asthma
*Brassica carinata*	Brassicaceae	Gomen	Leaf/seed as food; oil	Skin rush
*Brassica nigra*	Brassicaceae	Sinafich	Used as spice	Abdominal pain
*Capsicum annum *	Solanaceae	Qaria	Fruit used as spice	Vomiting, dysentery
*Carissa spinarum*	Apocynaceae	Agam	Edible fruit	Evil eye
*Coffea arabica*	Rubiaceae	Bunna	As stimulant	Wound
*Eragrostis tef*	Poaceae	Teff	Human food	Bone fracture
*Ficus sur*	Moraceae	Shoal	Edible fruit	Dysentery
*Foeniculum vulgare*	Brassicaceae	Ensilal	Used as spice	Abdominal pain
*Guizotia abyssinica*	Asteraceae	Nug	Sources of oil and fodder	Leech
*Hordeum vulgare*	Poaceae	Gebis	Food and fodder	Gastritis
*Linum usitatissimum*	Linaceae	Telba	Oil crop and fodder	Gastritis
*Lepidium sativum *	Brassicaceae	Feto	Used as spices/food	Diarrhea
*Lupinus albus *	Fabaceae	Gibito	Seed used as food	Hypertension
*Nigella sativa*	Apiaceae	Tiqur-azmud	Used as spice	Common cold, asthma
*Prunus persica *	Rosaceae	Kok	Edible fruit	Diarrhea
*Rhamnus prinoides*	Rhamnaceae	Gesho	Stimulant/spices	Leech, tonsils, skin infection
*Ruta chalepensis*	Rutaceae	Tila-adam	Seed/leaf as spices	Stomachache
*Urtica simensis*	Urticaceae	Samma	Edible leaf	Gastritis
*Zingiber officinale*	Zingiberaceae	Zinjibl	Used as spice	Stomachache

**Table 10:** Ranking of threats on medicinal plants cited by 7 respondents (values 1–5: 1 = the least destructive and 5 = the most destructive) (a single respondent mentioned two or more threats).

Threats	Respondents (R_1_–R_7_)							Total	%	Rank
	R_1_	R_2_	R_3_	R_4_	R_5_	R_6_	R_7_			
Overgrazing	5	4	4	5	4	4	3	29	26.12	1st
Charcoal and firewood	4	4	5	4	3	3	3	26	23.42	2nd
Deforestation	4	4	4	4	3	4	3	26	23.42	2nd
Drought	3	3	3	2	1	2	2	16	14.41	4th
Mining	3	2	3	2	1	1	2	14	12.61	5th
